# Radial Artery Reconstruction After Iatrogenic Cannulation-Related Occlusion: A Case Report

**DOI:** 10.7759/cureus.58214

**Published:** 2024-04-13

**Authors:** Christine V Schaeffer, Hayden L Cooke, Paul A Ghareeb

**Affiliations:** 1 Department of Plastic and Reconstructive Surgery, University of Kentucky College of Medicine, Lexington, USA; 2 Department of Orthopedic Surgery, Emory University School of Medicine, Atlanta, USA; 3 Department of Plastic and Reconstructive Surgery, Emory University School of Medicine, Atlanta, USA

**Keywords:** radial artery reconstruction, ischemic gangrene, upper extremity revascularization, digital necrosis, radial artery occlusion (rao)

## Abstract

A 59-year-old male, with a history of angiogram via the left radial artery during the workup for multi-trauma, presented to the hand clinic with a 14-day history of progressive critical ischemia in the left thumb and index finger, along with dry gangrene of the distal index fingertip. Radial artery occlusion was confirmed on imaging. The patient underwent radial artery thrombectomy, arterial reconstruction with vein graft, and amputation of the index fingertip. Postoperatively, perfusion to the thumb and index finger was restored, resulting in the resolution of associated pain and hypersensitivity. This case demonstrates the delayed presentation of ischemia following radial artery cannulation, which was successfully managed with radial artery thrombectomy and a saphenous vein graft.

## Introduction

Radial artery thrombosis and iatrogenic hand ischemia are known complications of radial artery cannulation. The reported rate of radial artery flow reduction or thrombosis following decannulation is 25%-33%. However, the rate of clinical hand ischemia in this setting is 0.09%-0.2% [1-3]. Initial management includes anticoagulation and vasodilators, followed by procedural intervention if medical management fails.

The literature on outcomes of digital ischemia following radial artery cannulation is limited to case series. Procedural interventions are not consistently successful, with a significant number of patients ultimately undergoing digital amputation. Additionally, time to surgery from diagnosis of ischemia is not reported [1,3,4]. Current literature lacks a gold standard for procedural intervention and time to intervention for digital ischemia following radial artery cannulation.

We present a case of critical ischemia affecting the index finger and thumb following radial artery cannulation for angiography. Perfusion was restored, and the pain was resolved with open thrombectomy and arterial reconstruction with a saphenous vein graft. This case demonstrates the successful salvage of digital ischemia in a patient with delayed presentation.

## Case presentation

A 59-year-old right-hand-dominant male presented to the emergency department following a motorcycle collision. His past medical history included type 2 diabetes, hypertension, and renal cell carcinoma (status post nephrectomy), and his social history included cigar smoking once a month. He sustained a grade IV splenic laceration, grade II kidney laceration, and left rib fractures. The left radial artery was accessed for splenic and renal angiography, which showed no evidence of active bleeding, pseudoaneurysm, or arterial irregularity. He underwent video-assisted thoracoscopic surgery (VATS) evacuation of left hemothorax, chest tube placement, and open reduction and internal fixation of left rib fractures four through eight for flail chest and respiratory failure. Eleven days following the initial presentation, the patient developed numbness, pain, and ecchymosis of the left thumb and index fingers. There was concern for occlusion of the radial artery with ischemia of the thumb and index finger. Aspirin 81 mg and Nitropaste were recommended twice daily for the affected fingers.

**Figure 1:**
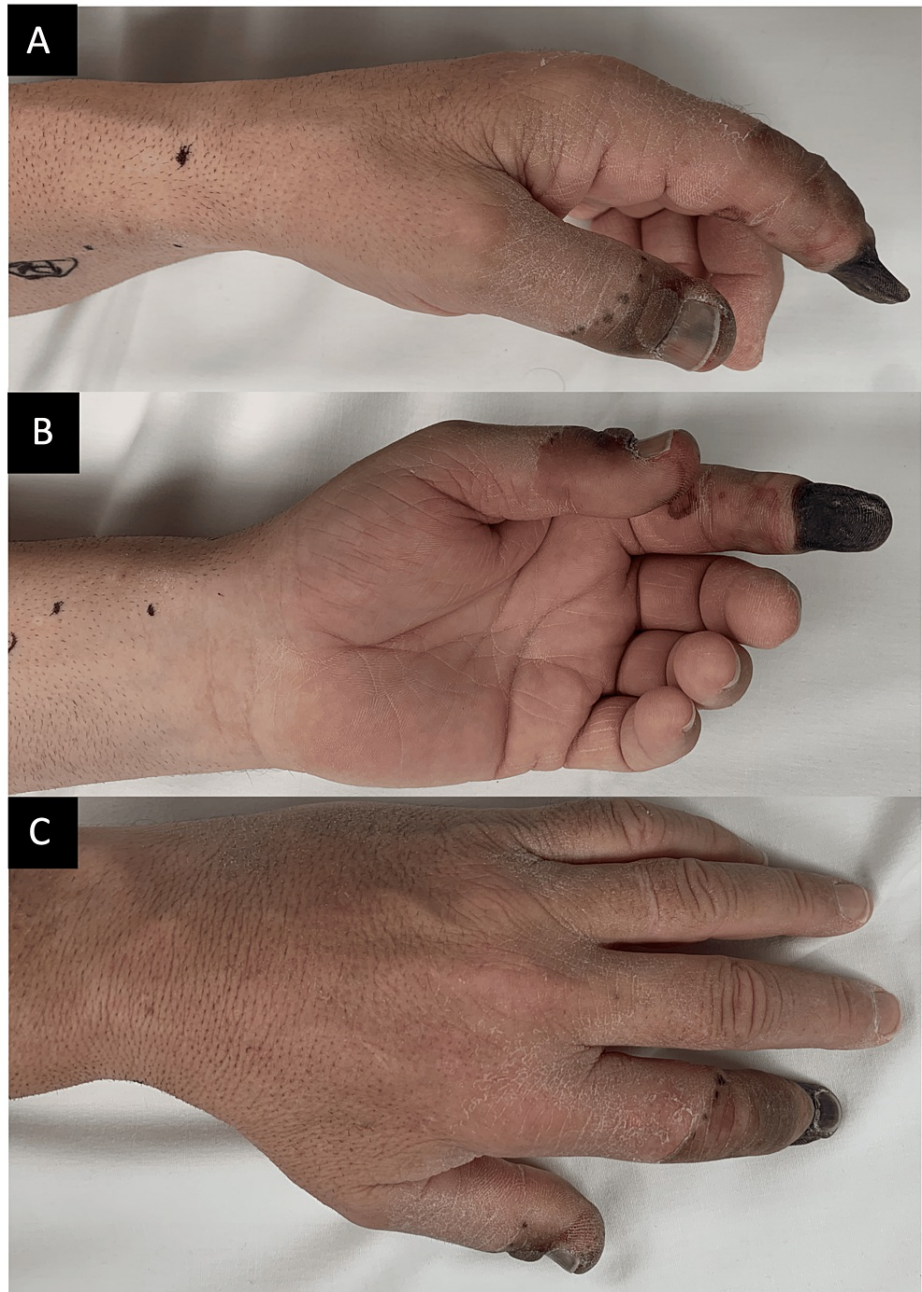
Threatened distal left thumb with mottling of the skin and distal dry gangrene of the left index finger 3.5 weeks following left radial artery cannulation: (A) volar, (B) oblique, and (C) dorsal.

The patient presented to the hand clinic with a 14-day history of ischemia in the left index finger and thumb. He reported progressive dark discoloration and cool temperature of the index finger and thumb in addition to significant pain and sensitivity to touch. On exam, the left ulnar artery was palpable with the absence of a radial pulse. Allen’s test was positive for inadequate flow from the radial artery. The thumb displayed mottling and felt cool up to the interphalangeal joint level. There was dry gangrene of the distal index finger to the level of the distal interphalangeal joint, with mottling to the level of the proximal interphalangeal joint (Figure 1). A computerized tomography angiogram (CTA) demonstrated occlusion of the radial artery in the proximal forearm, with distal reconstitution at the level of the palmar arch. Management options for thumb and index finger ischemia were discussed, including anticoagulation with observation to allow for tissue demarcation and radial artery reconstruction with vein graft as an attempt to salvage threatened tissue. The patient elected to proceed with radial artery reconstruction with vein graft and amputation of the gangrenous portion of the left index finger.

**Figure 2:**
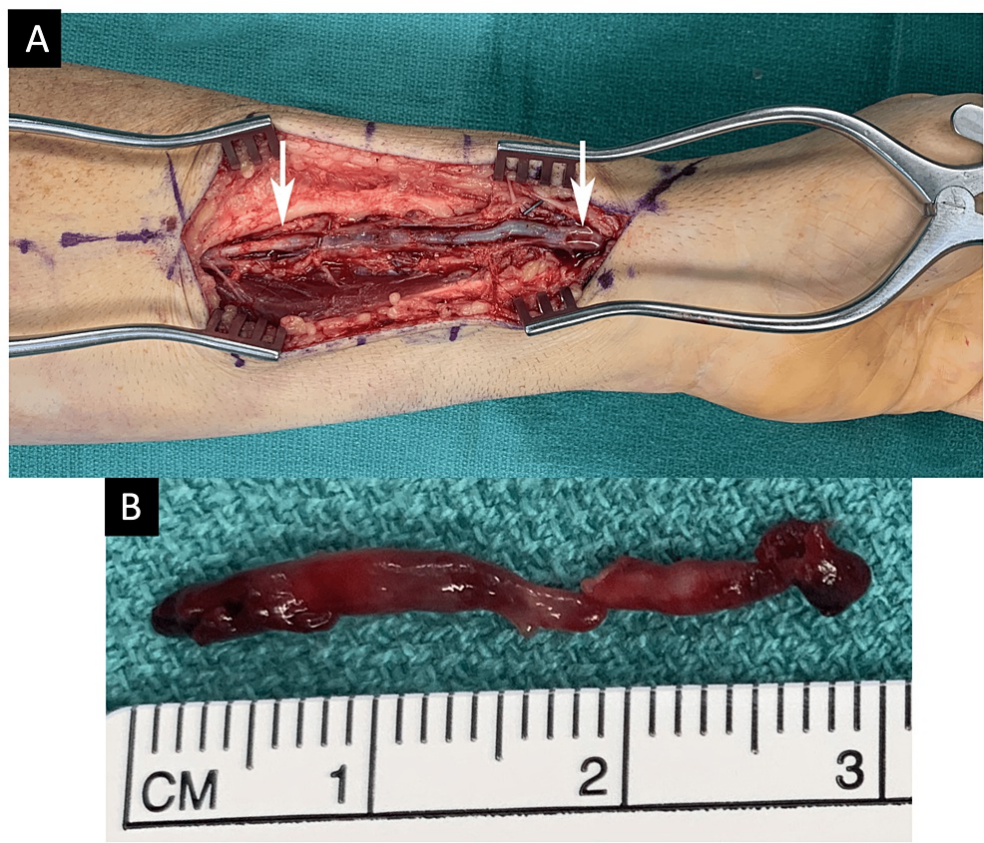
(A) Thrombosis of the left radial artery from the mid-forearm to the wrist (white arrows); (B) the artery was transected proximally with excision of the unhealthy vessel and distal thrombectomy.

**Figure 3:**
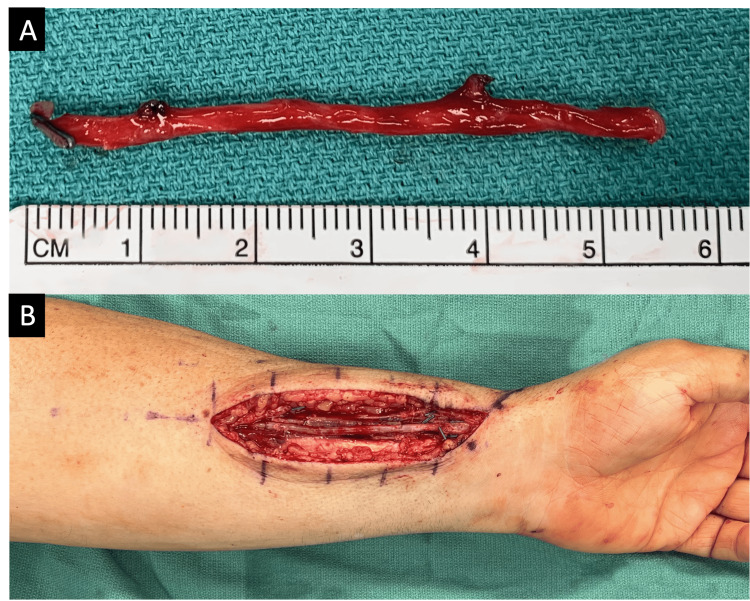
(A) Ipsilateral saphenous vein graft was harvested for arterial reconstruction; (B) end-to-end anastomosis of the reversed saphenous vein graft was performed with interrupted 8-0 nylon using the intraoperative microscope.

Intraoperatively, amputation of the gangrenous portion of the distal index finger was performed. Attention was turned to the forearm - the radial artery was noted to be thrombosed from the mid-forearm to the level of the wrist (Figure 2A). The artery was transected proximally at the level of a healthy-appearing artery. A thrombectomy was performed proximally with a size two French Fogarty catheter. No residual thrombus was noted from the proximal radial artery or brachial artery, and adequate inflow was confirmed. The distal radial artery was then transected, and a thrombectomy was performed distally with a size two French Fogarty catheter. The catheter was advanced 15-20 cm distally and retrieved a significant amount of thrombus (Figure 2B). Following thrombectomy, there was evidence of backfilling of the radial artery. A 6-cm saphenous vein graft was then harvested from the left thigh utilizing a standard microsurgical technique (Figure 3A). The vein was reversed and irrigated with heparinized saline. Proximal and distal bypass anastomoses were performed with 8-0 nylon suture in an interrupted fashion using the intraoperative microscope (Figure 3B). The arterial clamps were released proximally and distally, and flow was visible and palpable through the graft. The flow was confirmed with Doppler over the radial artery distal to the anastomosis and the Princeps pollicis branch of the radial artery. One- to two-second capillary refill was noted in the distal index finger and thumb. The patient was admitted overnight for observation and discharged on postoperative day 1 with therapeutic anticoagulation for two weeks.

**Figure 4:**
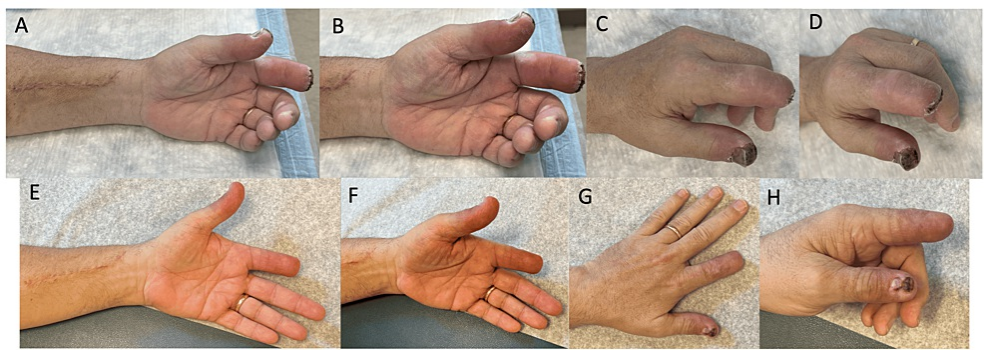
One-week postoperative follow-up (A, volar; B, oblique; C, dorsal; D, distal thumb) and five-week postoperative follow-up (E, volar; F, oblique; G, dorsal; H, distal thumb). The index finger amputation site healed without complication. (D) A 1 cm x 1 cm eschar of the distal tip of the thumb was noted at one week, and (H) it subsequently decreased significantly in size with local wound care by five weeks.  All fingers and thumb are pink with one- to two-second capillary refill, and radial pulse is palpable on examination.

The patient followed up at one week (Figures 4A-4D), two weeks, and five weeks post-op (Figures 4E-4H). He experienced complete resolution of rest pain and hypersensitivity in the thumb and index finger at his first postoperative appointment. The index finger amputation site healed without complication. He developed a 1 cm x 1 cm eschar of the distal tip of the thumb, which healed with local wound care (Figures 4D, 4H). His fingers remained pink with one- to two-second capillary refill, and radial pulse was palpable on examination.

## Discussion

The incidence of iatrogenic hand ischemia following radial artery cannulation is a rare but devastating complication [3]. The primary arterial supply to the thumb and index finger originates from the radial artery, first palmar metacarpal artery, or first dorsal metacarpal artery [5]. There is significant variability in the anatomy of the superficial and deep palmar arches - anatomic studies demonstrated complete superficial palmar arch to be present in 43% to 97% of hands [6-9]. The proposed etiologies of digital ischemia following radial artery cannulation include occlusion of the radial artery in the setting of an incomplete superficial palmar arch, radial artery and superficial palmar arch occlusion, and/or digital embolization of the radial artery thrombus [5,10].

Assessment of the collateral circulation to the hand is commonly performed before radial artery cannulation. Techniques include modified Allen’s test, Doppler ultrasound, and pulse oximetry. Several studies have shown that these assessment tools are poor predictors of ischemic changes following radial artery cannulation [1]. Previous studies have attempted to identify factors that increase the risk of hand ischemia following radial artery cannulation, including concomitant hypotension, high-dose vasopressor administration, preexisting vascular disease of the cannulated upper extremity, female sex, number of arterial puncture attempts, and catheter size/length. However, these factors have also not been found to be reliable predictors for ischemia after radial artery cannulation [1,3,11,12].

Physical examination findings concerning hand ischemia after radial artery cannulation include the absence of palpable radial pulse, mottling/discoloration of the skin, paresthesia, and painful and/or cold fingers [1,3,10]. Imaging studies to assess arterial inflow include arteriography - CTA versus standard angiogram versus magnetic resonance angiography (MRA) - and duplex Doppler studies [1,5,10]. If imaging demonstrates an occlusive thrombosis, the management options include anticoagulation, catheter-directed thrombolysis, embolectomy with Fogarty catheter, open thrombectomy with patch angioplasty, or arterial reconstruction with vein graft [5,11,13]. Saphenous vein grafts, as used in this case, can be harvested up to 70 cm in length [14]. Newer strategies to mitigate morbidity associated with upper extremity ischemia in this patient population have included the injection of Botulinum toxin into the hand. Botulinum toxin injections have been utilized in the treatment of digital ischemia associated with the Raynaud phenomenon, resulting in improved digital oxygen saturation and perfusion [15]. Laarakker and Borah recently described the application of this therapeutic strategy in patients with acute hand injuries, including radial artery catheterization, in a small case series and found Botulinum injections to increase digital salvage rates [16].

Outcomes in this patient population are limited to case series in the literature. In a recently published review of the available literature on ischemia following radial artery catheterization, Ying et al. reported that of the 57 patients diagnosed with hand ischemia, 20 (35%) went on to require digital amputation [3]. Valentine et al. reported a retrospective eight-patient case series of patients diagnosed with radial cannula-induced thrombosis and subsequent ischemia (Stage IIB - defined as *immediately threatened* with rest pain or motor loss) [1,4]. Five patients underwent operative intervention with arterial thrombectomy - four had patch angioplasty, and one had vein interposition grafting. Three patients were managed non-operatively - two patients were treated with vasodilators (intra-arterial verapamil and intravenous diltiazem versus oral diltiazem) and anticoagulation (low-dose intravenous heparin), and one patient was observed without medical treatment [1]. The outcomes for patients who underwent operative revascularization included one postoperative patient death, thrombosis of 75% of patch angioplasty repairs within 24 hours, and all surviving patients went on to develop gangrene of first or second digit - 50% underwent digital amputation [1]. In the non-operative group, only the patient managed with observation alone went on to develop digital ischemia [1]. Time to surgery from diagnosis of hand ischemia was not reported in this series. Based on these outcomes, the authors hypothesized that digital gangrene was a result of distal embolization from the radial artery, and therefore, radial artery revascularization would not prevent the progression of digital ischemia [1]. In contrast to these previously reported results, our patient presented with Stage IIB ischemia of the thumb and proximal index finger (Stage IIB ischemia), which were successfully reperfused following radial artery thrombectomy and saphenous vein grafting.

## Conclusions

Hand ischemia is a rare complication of radial artery cannulation. However, patients diagnosed with ischemia in this clinical setting have high rates of subsequent digital gangrene and amputations. The literature lacks a gold standard procedural intervention for digital ischemia in the setting of radial artery occlusion following cannulation. Additionally, there is no guidance in the literature regarding the time to intervention and the potential impact on outcomes. We report a case of a patient with a two-week history of threatened digits (thumb and index), which were successfully reperfused following radial artery thrombectomy and saphenous vein grafting. Further studies are required to delineate a standardized approach to the workup and management of iatrogenic hand ischemia following radial artery cannulation. 
